# High-Performance Wireless Ammonia Gas Sensors Based on Reduced Graphene Oxide and Nano-Silver Ink Hybrid Material Loaded on a Patch Antenna

**DOI:** 10.3390/s17092070

**Published:** 2017-09-09

**Authors:** Bian Wu, Xingfei Zhang, Beiju Huang, Yutong Zhao, Chuantong Cheng, Hongda Chen

**Affiliations:** 1 National Key Laboratory of Antennas and Microwave Technology, Shaanxi Joint Key Laboratory of Graphene, Xidian University, Xi’an 710071, China; xingfeizhang2014@163.com (X.Z.); ytzhao@stu.xidian.edu.cn (Y.Z.); 2 State Key Laboratory on Integrated Optoelectronics, Institute of Semiconductors, Chinese Academy of Sciences, Beijing 100083, China; chengchuantong@semi.ac.cn (C.C.); hdchen@semi.ac.cn (H.C.)

**Keywords:** ammonia sensor, reduced graphene oxide (rGO), Ag ink, patch antenna

## Abstract

Reduced graphene oxide (rGO) has been studied as a resistive ammonia gas sensor at room temperature. The sensitive hybrid material composed of rGO and nano-silver ink (Ag-ink) was loaded on a microstrip patch antenna to realize high-performance wireless ammonia sensors. The material was investigated using scanning electron microscopy (SEM) and X-ray photoelectron spectroscopy (XPS). Firstly, interdigital electrodes (IDEs) printed on the polyethylene terephthalate (PET) by direct printing were employed to measure the variation of resistance of the sensitive material with the ammonia concentration. The results indicated the response of sensor varied from 4.25% to 14.7% under 15–200 ppm ammonia concentrations. Furthermore, the hybrid material was loaded on a microstrip patch antenna fabricated by a conventional printed circuit board (PCB) process, and a 10 MHz frequency shift of the sensor antenna could be observed for 200 ppm ammonia gas. Finally, the wireless sensing property of the sensor antenna was successfully tested using the same emitted antenna outside the gas chamber with a high gain of 5.48 dBi, and an increased reflection magnitude of the emitted antenna due to the frequency mismatch of the sensor antenna was observed. Therefore, wireless ammonia gas sensors loaded on a patch antenna have significant application prospects in the field of Internet of Things (IoTs).

## 1. Introduction

Ammonia (NH_3_) has been widely used in the chemical industry, the light industry, chemical fertilizer, pharmaceutical, synthetic fiber, etc. Characterized with an irritating odor, it stimulates the eyes and skin rapidly at even a very low concentration and causes lung damage or even death with a large amount of inhalation [1]. Therefore, it is essential to develop ammonia sensors with high sensitivity and operability at room temperature. Currently, most conventional ammonia sensors are based on metal oxides, such as tungsten trioxide [2], zinc oxide [3], titanium oxide [4], and indium oxide [5]. These sensors usually suffer from a high price, a high working temperature (200–350 °C), and a short service life, necessitating the development of a novel ammonia sensor that overcomes these disadvantages. There has been significant work done in wireless CNT-based gas sensors that rely on detecting either change in the resonant frequency or change in the amplitude upon exposure to the analyte of interest, and the method relying on the shift of the resonant frequency is the most effective for remote sensing. However, for long distance wireless detection with two antennas, the frequency shift of another antenna does not vary significantly as the amplitude changes. An ammonia gas sensor is presented utilizing carbon nanotubes (CNTs) integrated with a microstrip patch antenna on a thin paper substrate and utilizes the shift in resonant frequency as a means of gas detection [6]. Such an ammonia sensor could also be enabled by the advent of graphene, a single-atom layer carbon material, and exhibits exceptional and intriguing properties, such as high conductance, mechanical strength, and large specific surface [7].

Recent research has shown that the composite of reduced graphene oxide (rGO) with other materials enhances sensing abilities [8,9,10,11,12,13]. Compared with single-layer graphene fabricated by the chemical vapor deposition (CVD) method, rGO has the potential to be preferred for gas sensors because of its lower cost and larger production quantity [9]. Moreover, it left oxygen-functional groups that provide gas molecules with an increased amount of adsorption sites [12,13]. Until now, most previous ammonia sensors using rGO as a part of sensitive materials are focused on resistance or current variation, unable to realize wireless sensing. Recently, some studies have realized wireless sensing using a coil antenna [14], a substrate integrated waveguide (SIW) slot antenna [15], and a dipole antenna [16]. In [14], a wireless sensing system is proposed using integrated arrays of field-effect transistors, and sensors comprising graphene channels and silver nanowire electrodes, interconnected with a wireless antenna. This wireless sensing system can only detect harmful gases at a distance of 2 mm. Moreover, in [15], two SIW-based cavity resonators, a ring-slot resonator and a complementary split ring resonator (CSRR), were fabricated and coated with CVD-grown graphene. Graphene covers the gaps in the radiation energy, so the energy cannot radiate effectively and wireless sensing cannot be realized remotely. In [16], radio frequency identification (RFID)-based wireless sensor system is fabricated using a carboxyl group functionalized polypyrrole (C-PPy) nanoparticles (NPs). The gain of the dipole antenna is not high, so it is not good enough to detect effectively. Despite their improved performance, these sensors are still more or less deficient, with disadvantages such as low gain, unsatisfactory long-distance transmission, hard integration, wide working frequency, and inefficient detection. Therefore, ammonia sensors with further enhanced sensing abilities are still strongly required.

In this paper, we propose wireless NH_3_ sensitive materials composed of rGO and nano-silver ink (Ag-ink) loaded on a high-gain microstrip patch antenna. The hybrid materials were investigated by scanning electron microscopy (SEM) and X-ray photoelectron spectroscopy (XPS). The dynamic response and sensitivity of the sensor were tested by the source meter and its corresponding software, while the wireless sensing performance of the sensor material load on the patch antenna was measured by a vector network analyzer at room temperature (25 ± 2 °C). The radiation characteristics of the sensitive antenna were tested with an antenna measuring system.

## 2. Synthesis and Characterization

### 2.1. Synthesis of Reduction Graphene Oxide

Graphene oxide (GO) was synthesized from natural graphite powder using a modified Hummer’s method [17]. Briefly, 0.5 g of high purity graphite powder and 0.74 g of NaNO_3_ were placed into 34 mL of H_2_SO_4_ (98%) and vigorously stirred in an ice-water bath. Then, 5.0 g of KMnO_4_ was later slowly added, and it was ensured that the temperature did not exceed 20 °C during the process. The mixture was continuously stirred for 3 h at 35 °C. Next, 250 mL of water and 4 mL of H_2_O_2_ (30 wt %) were slowly added. The obtained bright yellow suspension was washed 5 times with HCl and water (1:10 v:v). The solid (GO) was finally dried at 50 °C over a 24 h period. Finally, 50 mg of the as-prepared GO were dispersed into 50 mL of DI water by sonicating for 1 h. Then, 50 μL of hydrazine monohydrate was added, and the mixture was heated at 95 °C for 1 h. After centrifugation and washing with water and ethanol several times, the obtained rGO was re-dispersed in 50 mL of deionized water by sonication for 1 h.

### 2.2. Fabrication of Sensor Devices with rGO/Ag-ink

**Figure 1:**
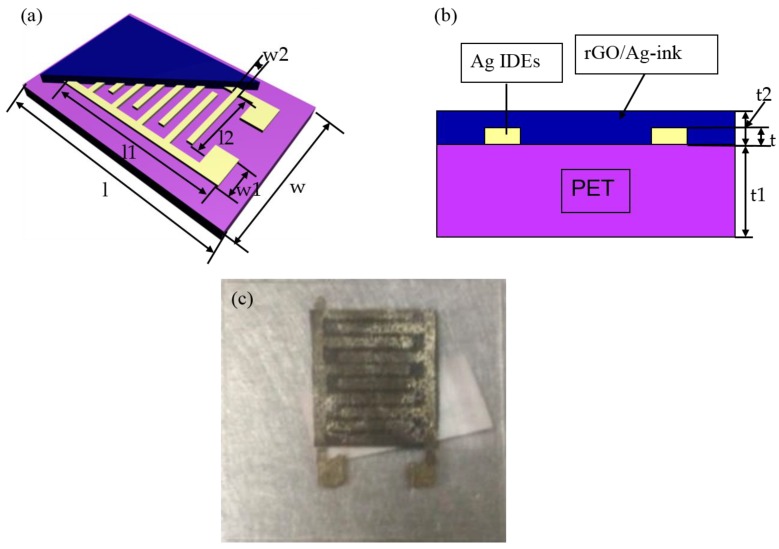
Schematic and physical map of the coated interdigital electrodes (IDEs) with reduced graphene oxide (rGO)/nano-silver ink (Ag-ink). (**a**) Schematic diagram; (**b**) cross-sectional view; (**c**) microscopic picture of the coated IDEs.

**Figure 2:**
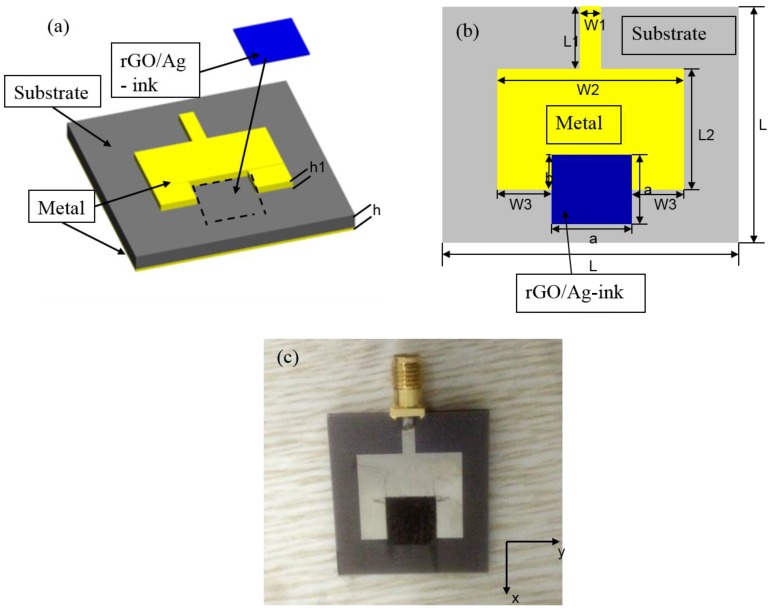
Schematic and physical map of the microstrip patch antenna with rGO/Ag-ink. (**a**) Schematic diagram; (**b**) top-sectional view; (**c**) microscopic picture of the coated microstrip patch antenna with rGO/Ag-ink.

In the paper, two kinds of devices were fabricated to test the sensing performance of rGO/Ag-ink. In Figure 1, Ag-ink was bought from a commercial company (KunShan Hisense Electronics CO, KunShan, China). Microplotter II (Middleton, WI, USA) was employed to print interdigital electrodes (IDEs) on a flexible PET and heated on the hot plot for 30 min at 145 °C. The dimensions of the proposed IDEs sensor with rGO/Ag-ink are given in Table 1. Figure 2 illustrates the proposed sensor antenna with rGO/Ag-ink, and the dimensions are given in Table 2. The patch antenna with a gap was fabricated by a conventional printed circuit board (PCB) process. More stable Teflon was used as the substrate of the patch antenna. Then, the volume feed ratio of Ag-ink to as-prepared rGO solution was set as 1:1 and sonicated for 30 min. For the last stage of fabrication, the mixture was placed in the designated gap area and sit for additional 24 h at room temperature.

### 2.3. Characterization of rGO/Ag-ink Hybrid Material

**Figure 3:**
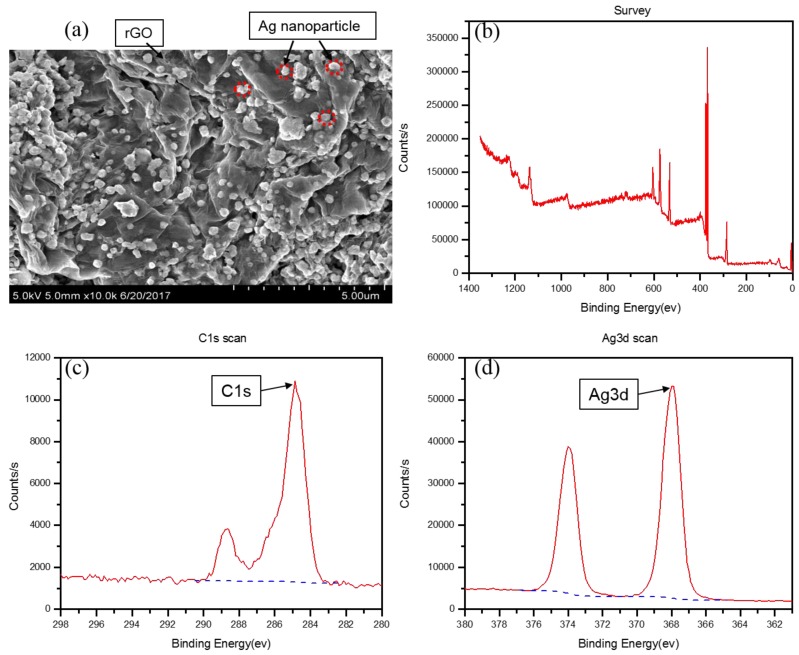
(**a**) Representative SEM images of rGO/Ag-ink films; (**b**) high-resolution C1s XPS spectra of rGO/Ag-ink films; (**c**) C 1s XPS spectra for rGO/Ag-ink; (**d**) Ag 3d XPS spectrum of rGO/Ag-ink.

The morphology of the rGO/Ag-ink film was studied using a field emission scanning electron microscope (SEM) (Hitachi S4800, Tokyo, Japan) in Figure 3. Figure 3a represents obvious fluctuant transparent rGO/Ag-ink films with dispersed Ag nanoparticles or their aggregation, which should be attributed to the supporting function of Ag nanoparticles under folded rGO sheets. Ag nanoparticles were well attached to rGO, increasing the conductivity and the surface area ratio of the rGO/Ag-ink film. The surface chemical composition was characterized by an X-ray photoelectron spectroscope (XPS) (Thermo escalab 250Xi, Waltham, MA, USA), as shown in Figure 3b. From the spectra, three sharp peaks at 282, 369 and 529 eV, representing the C 1s, Ag 3d and O 1s peaks, respectively, can be observed. Figure 3c exhibits the C 1s XPS spectra of the rGO/Ag-ink, which has two main peaks at 284.8 eV and 288.6 eV corresponding to the C–C and C=C species, respectively. Furthermore, the characteristic peak of Ag 3d doublet for Ag NPs is shown in Figure 3d. The Ag 3d 5/2 and Ag 3d 3/2 binding energies appear at 368.6 and 374.6 eV, respectively.

## 3. Experimental Results and Discussions

### 3.1. The Sensitivity of rGO/Ag-ink on IDEs

**Figure 4:**
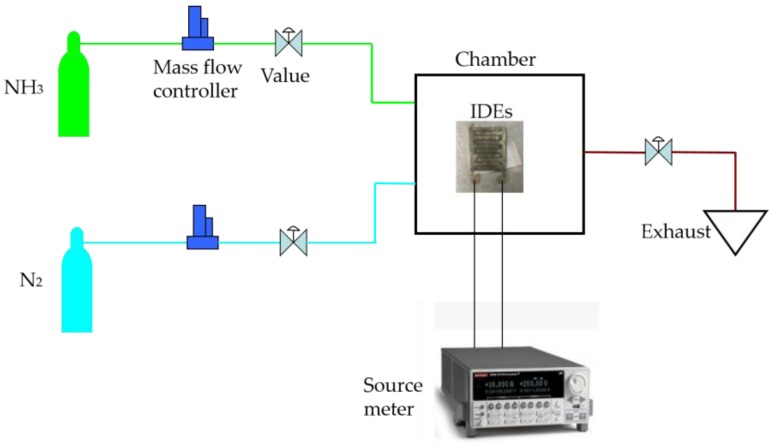
Scheme of ammonia gas characterization for IDEs sensor with rGO/Ag-ink.

**Figure 5:**
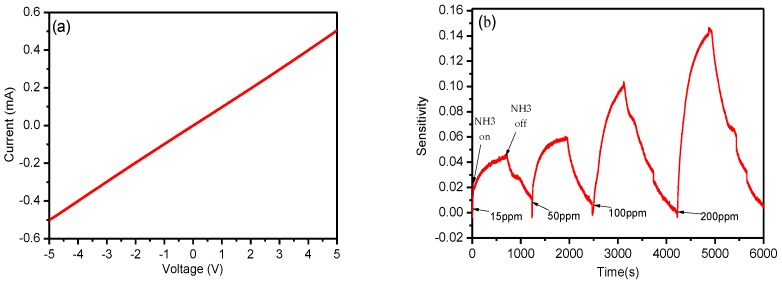
(**a**) Current–voltage (I-V) curves for the coated IDEs with rGO/Ag-ink; (**b**) dynamic response of the IDEs sensors to different concentrations of NH_3_ at room temperature.

In order to evaluate the dynamic response and sensitivity of the proposed rGO/Ag-ink sensor, the IDEs covered by the rGO/Ag-ink hybrid were placed into the self-made chamber and connected to the Keithley 2612A Source Meter (Figure 4). Before inletting ammonia, we used nitrogen to empty the air in the chamber. Because the amount of 200 ppm of ammonia is relatively small, the chamber pressure was almost constant. Successfully fabricated IDE sensors were tested for their initial resistance, which was measured to be 10.31 kΩ (Figure 5a). The sensor sensitivity was calculated by Equation (1):

(1) $$R\mathrm{esponse}=\frac{Rg-R_{0}}{R_{0}}$$

where Rg stands for the resistance of the sensor exposed to NH_3_ at room temperature; $R_{0}$ is the sensor resistance before the injecting gas. The response time and the recovery time of the gas sensor are two significant parameters defined as up to 90% and 10% of the final stable resistance.

The response of the IDEs sensor with rGO/Ag-ink with 15 ppm, 50 ppm, 100 ppm, and 200 ppm of NH_3_ was 4.25%, 6.1%, 10.08%, and 14.7%, respectively (Figure 5b). The prepared sensor showed a slightly higher response in a comparison with rGO modified by metal or metal oxide nanoparticle ammonia sensors [12,18]. In [12], for example, the author obtained a silver-nanoparticle-decorated rGO hybrid ammonia sensor, and the response of this sensor was 7.7 ± 0.2% at 2500 ppm NH_3_. Additionally, the ZnO nanoparticles/reduced graphene oxide bilayer thin film sensors with a response of 3.05% (50 ppm NH_3_) were obtained in [18]. From the diagram, the resistance of the sensitive component increases after injecting ammonia. This can be explained as follows: rGO is a p-type semi-conductor material. Silver nanoparticles, as the dominant active adsorption sites for NH_3_, strengthen the sensitive performance; ammonia, as a reducing gas, provides a large number of electrons. Ammonia is attached to the sensing material, and electrons are transferred from the ammonia to the RGO. The holes and electrons are offset against each other, causing an increase in the sensor's resistance.

### 3.2. The Reflection of the Patch Antenna with rGO/Ag-ink

**Figure 6:**
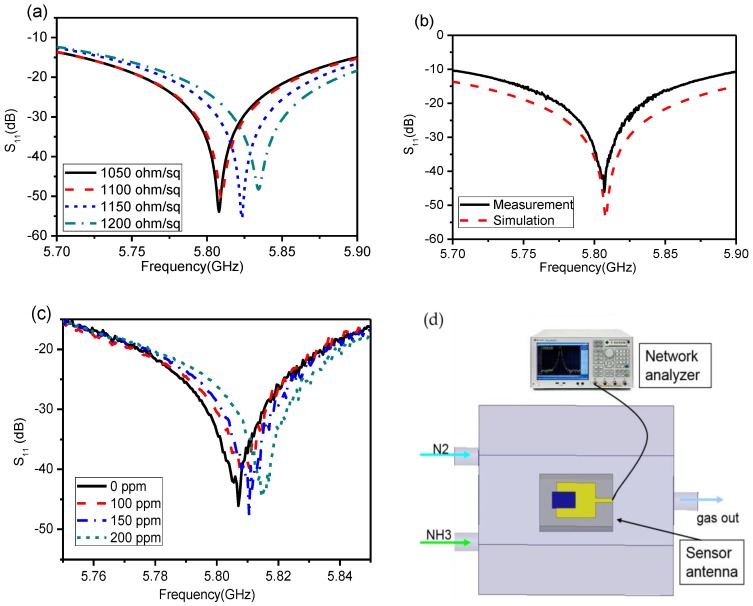
(**a**) Simulation reflection coefficients of the patch antenna loaded with rGO/Ag-ink hybrid material under different sheet resistances; (**b**) Measured and simulated reflection coefficient of the microstrip patch antenna loaded with rGO/Ag-ink hybrid material under 0 ppm ammonia; (**c**) Measured reflection coefficients of environmental gas sensors with different ammonia gas densities; (**d**) Schematic of the experimental set-up of the sensor antenna with rGO/Ag-ink that is connected to the network analyzer.

The sheet resistance of the film was tested with a four-probe method, which was close to 1000 ohm/sq. The microstrip patch antenna loaded with rGO/Ag-ink hybrid material was simulated by HFSS. In the simulation setup, the initial sheet resistance of the rGO/Ag-ink layer was 1050 ohm/sq, which is close to the measurement taken after the film growth. The frequency of the patch antenna loaded with rGO/Ag-ink sheets is designed to be 5.8 GHz for the proof of concept. It is worth noting that the sheet resistance of the rGO/Ag-ink material increases when it detects ammonia gas. The increase in rGO/Ag-ink sheet resistance will raise the resonant frequency of the antenna. Therefore, the rGO/Ag-ink sheet resistance Rs varies from 1050 to 1200 ohm/sq in the simulation set-up. The corresponding variation of resonant frequency is illustrated in Figure 6a, showing that the resonant frequency of the sensor antenna rises as the resistance of the rGO/Ag-ink sheets increases.

Next, we measured the reflection coefficients of the sensor antenna. The microstrip patch antenna with a sensitive component is placed in a homemade chamber and connected to the vector network analyzer (Agilent E5071B, Palo Alto, CA, USA) by the 50 ohm Sub-Miniature-A (SMA) connector, as shown in Figure 6d. Firstly, the reflection coefficient of the sensor antenna was tested in the absence of ammonia gas and compared with the simulation results, as shown in Figure 6b. Then, the reflection coefficients of the antenna under different concentrations of ammonia could be measured. The measurement results of the two devices are plotted in Figure 6c, illustrating that the resonant frequencies increase gradually with the concentration of ammonia. Before injecting ammonia, the measured S_11_ peak point of the antenna is −42.7 dB and its frequency is 5.806 GHz. Then, the peak points of the antenna moved to 5.808 GHz, 5.811 GHz, and 5.816 GHz, after injecting 100 ppm, 150 ppm, and 200 ppm ammonia, respectively. The variation of ammonia concentration in the test has a similar function with the impedance change in the simulation, and the sheet resistance of the film is increased by the use of ammonia, which affects the resonant frequency of the antenna. The sensitive material transferred on the patch antenna can be seen as the load impedance. The S11 parameter of the antenna can be calculated by the following equation:

(2) $$S_{11}=20\log\left|\frac{Z_{Load}-Z_{1}{}^{*}}{Z_{Load}+Z_{1}}\right|$$

where $Z_{Load}$ represents the load impedance, and $Z_{1}$ represents the self-impedance of sensor antenna, with * being its complex conjugate. After injecting ammonia gas, the resistance of the p-type graphene sensitive material increases; as a result, the impedance of the antenna changes accordingly, resulting in the frequency shift of the S11 parameter, as illustrated in Figure 6a. Therefore, we can infer that the increase in the antenna’s resonant frequency is due to the increase of ammonia concentration.

### 3.3. Wireless Sensing Using Patch Antennas with rGO/Ag-ink

**Figure 7:**
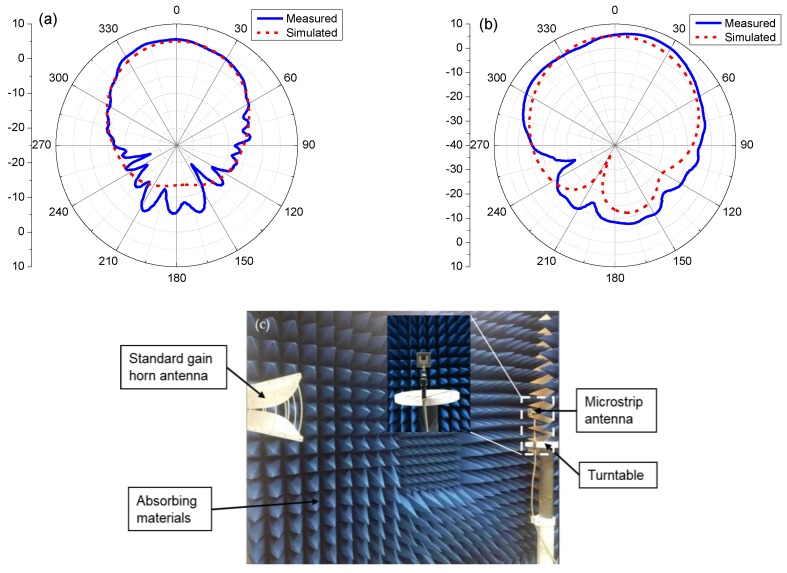
(**a**) Measured (solid line) and simulated (dotted line) radiation patterns of the patch antenna loaded with rGO/Ag-ink sheets in the azimuth plane (yoz); (**b**) Measured (solid line) and simulated (dotted line) radiation patterns of the patch antenna loaded with rGO/Ag-ink in the elevation plane (xoz); (**c**) The antenna measuring system in the anechoic cabinet using the turntable included in the system.

The radiation pattern of the patch antenna was measured with the antenna measuring system in the anechoic cabinet using the turntable included in the system. Figure 7 shows that the measured (solid line) and simulated (dotted line) radiation patterns of the antenna are in good agreement with the main lobe, and the measured maximum gain is 5.48 dBi at φ = 0° and θ = 0°.

**Figure 8:**
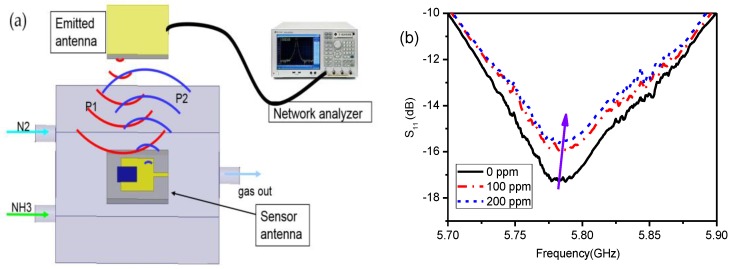
Wireless sensor monitoring of ammonia gas. (**a**) Schematic diagram of the wireless gas sensing set-up with two patch antennas loaded with rGO/Ag-ink sheets; (**b**) The measured S_11_ of the emitted antenna with various ammonia concentrations. The reflection magnitude decreases while the resonant frequency slightly increases as the ammonia concentration increases.

Two identical patch antennas loaded with rGO/Ag-ink material were illustrated to characterize wireless sensing performance. The emitted antenna was connected to a vector network analyzer (VNA), while the sensor antenna was set inside the gas chamber connected to the standard load impedance via an SMA connector. Two antennas were placed face to face with a distance between them of about 200 mm, as shown in Figure 8a. The antenna connected to the VNA emitted an interrogation signal (P1), and the sensor antenna then induced the interrogation signal, activating a reflected signal (P2) (backscattering) [19,20]. Its power was calculated by the FRIIS transmission equation [21,22]:

(3) $$P_{r}=P_{t}G_{t}G_{r}{\left(\frac{\lambda }{4\pi r}\right)}^{2}$$

where $P_{t}$ is the power, $G_{t}$ is the gain of the emitted antenna, $G_{r}$ is the gain of the sensor antenna, r represents the distance of the two antennas, and λ stands for the operating wavelength.

Since the sensor antenna becomes mismatched with the emitted antenna as the ammonia concentration changes, part of the induced power of the sensor antenna is reflected back to the emitted antenna:

(4) $$P_{ref}=P_{r}\eta$$

where η is the power reflection coefficient of the sensor antenna. The backscattered power received by the emitted antenna from the sensor antenna can be calculated by

(5) $$P_{backscatter}=P_{ref}G_{t}G_{r}{\left(\frac{\lambda }{4\pi r}\right)}^{2}=P_{t}G_{t}^{2}G_{r}^{2}\eta {\left(\frac{\lambda }{4\pi r}\right)}^{4}.$$

The resonant frequency of the sensor antenna varied with different concentrations of ammonia gas, so the backscattered power is adjusted. As a result, the reflection magnitude of the emitted antenna changed accordingly. Figure 8b shows the reflection variation of the emitted antenna with different concentrations of ammonia. The wireless test results can be explained by the same frequency resonance theorem [23,24]. We noticed that the resonant frequency of the emitted antenna is 5.76 GHz, while that of the sensor antenna is 5.8 GHz. Since the resonant frequency of the sensor antenna obviously increases with the ammonia concentration, as depicted in Figure 6c, the magnitude of the reaction changes according to Equation (5), which leads to an increased reflection magnitude of emitted antenna due to the frequency mismatch of the two antennas.

## 4. Conclusions

In this paper, a novel wireless ammonia sensor loaded on a microstrip patch antenna operating at a microwave frequency with rGO and nano-silver ink as a hybrid sensing material was proposed, demonstrating a low-cost and feasible method of gas detection. Combining an ammonia sensor with a high gain patch antenna, which can detect different concentrations of ammonia while maintaining the characteristic of the patch antenna itself, is a creative idea. Compared with coil antennas and dipole antennas, the patch antenna has more advantages with a wireless sensor, as shown in Table 3. More importantly, it will detect toxic gases effectively, while avoiding potential harm to the human body. In other words, long-distance wireless sensor detection can be achieved. Thus, the proposed devices will be an indispensable component for Internet of Things (IoTs).

## Figures and Tables

**Table 1:** Dimensions of the proposed IDEs sensor with rGO/Ag-ink.

Parameters	Values (mm)	Parameters	Values (mm)	Parameters	Values (mm)	Parameters	Values (mm)	Parameters	Values (mm)
**l**	30	l1	15	l2	12	t	0.0005	t2	0.0015
**w**	20	w1	5	w2	0.5	t1	0.25		

**Table 2:** Dimensions of the proposed sensor antenna with rGO/Ag-ink.

Parameters	Values (mm)	Parameters	Values (mm)	Parameters	Values (mm)	Parameters	Values (mm)	Parameters	Values (mm)
L	30	L2	13.8	W2	20	a	9.5	h	1.9
L1	8	W1	2.5	W3	5.25	b	6.75	h1	0.017

**Table 3:** Properties of the proposed wireless gas sensor antenna and other sensor antennas.

Sensor Type	Gain Level	Integration Level	Identification Range	Operation Bandwidth	Reference
**Patch Antenna**	high	easy	long	narrow	This work
**Coil Antenna**	low	hard	short	wide	[14]
**SIW Slot Antenna**	low	easy	short	wide	[15]
**Dipole Antenna**	average	hard	average	wide	[16]
